# Health beliefs before and after participation on an exercised-based rehabilitation programme for chronic knee pain: Doing is believing

**DOI:** 10.1186/1471-2474-11-31

**Published:** 2010-02-11

**Authors:** Michael V Hurley, Nicola Walsh, Vanita Bhavnani, Nicky Britten, Fiona Stevenson

**Affiliations:** 1 Rehabilitation Research Unit, Kings College London, London, UK; 2 Faculty of Health Life Sciences, University of the West of England, Bristol, UK; 3 Department of General Practice & Primary Care, Kings College London, London, UK; 4 Institute of Health Service Research, Peninsula Medical School, University of Exeter, UK; 5 Department of Primary Care and Population Health, UCL Medical School, London, UK

## Abstract

**Background:**

To explore the health beliefs, experiences, treatment and expectations of people with chronic knee pain, and investigate if, how and why these change after taking part on an integrated exercise-based rehabilitation programme - *Enabling Self-management and Coping with Arthritis knee Pain through Exercise, ESCAPE-knee pain*.

**Methods:**

Semi-structured interviews were conducted with people with chronic knee pain, before (n = 29) and after (n = 23) participation on the programme. Thematic analysis was used to document people's baseline health beliefs, attitudes and cognitions, and to see if how and why these changed after completing the programme.

**Results:**

Initially people had poor understanding and negative, fatalistic beliefs about the management or prognosis for knee pain. Following the programme the majority of participants had positive experiences describing improvement in pain, physical and psychosocial functioning, greater knowledge and understanding of their condition and treatment options, and in their ability to use exercise to control symptoms. Beliefs about the causation and prognosis of knee pain were unchanged, but their concerns about possible dangers of exercise had decreased, they appreciated how exercise could reduce symptoms (treatment beliefs) and their confidence in their ability to use exercise to effect improvements (exercise self-efficacy) increased. These improvements were attributed to the content and structure of the programme, and the care and guidance of the physiotherapist. Several expressed a need for on-going support.

**Conclusions:**

*ESCAPE-knee pain *appears to achieve improvements by increasing people's treatment belief in safety and the utility of exercise to control symptoms, rather than alteration in their beliefs about causation or prognosis.

**Trial Registration:**

Current Controlled Trials ISRCTN94658828

## Background

Chronic knee pain is a major cause of disability [1-4]. It is commonly perceived to be an inevitable, incurable consequence of ageing, and managed with medication and surgery [5-10]. In fact, self-management programmes [11-14] and exercise [15,16] improve physical and psychosocial health and wellbeing. These interventions are usually delivered separately, but integrating self-management strategies with active participation on an exercise regimen might enhance the separate effects [17,18].

We devised a rehabilitation programme, *Enabling Self-management and Coping with Arthritic knee pain through Exercise (ESCAPE-knee pain)*, that integrates an exercise regimen with education and teaches participants simple coping and self-management strategies and skills. The programme aims to increase people's understanding of their condition, challenge inappropriate beliefs, advise them what (not) to do, and successful completion of a challenging exercise regimen enables participants to appreciate their capabilities and the tangible benefits of regular physical activity. The programme's underlying premise is that coupling better understanding with positive experience of exercise will improve beliefs in relation to the safety and value of exercise in the management of chronic knee pain (their treatment beliefs of exercise), and enhance their confidence in their ability to implement exercise as a self-management strategy (their exercise self-efficacy).

The programme applied Leventhal's self-regulation model of illness [19], which posits people's understanding of illness is based on their rationalisation about its cause, consequences, timeline, trajectory, prognosis and the ability of them or others to successfully treat (control or cure) the condition [19]. These cognitions are acquired from people's beliefs, experiences and information gleaned from family, friends, healthcare practitioners and the media. Importantly, the self-regulation model is malleable, so positive or negative experiences and information can alter people's illness beliefs, coping strategies and behaviour [19]. Therefore, interventions that address people's maladaptive beliefs and behaviour, might improve self-regulation of pain, disability, physical and psychosocial well-being [20].

In a randomised controlled trial (RCT) *ESCAPE-knee pain *significantly improved self-reported physical functioning, pain, anxiety, depression, exercise health beliefs and exercise self-efficacy, but had little effect on physiological variables, such as knee muscle strength [21]. Thus successful rehabilitation seems to depend more on improving psychosocial well-being and health beliefs than physiological improvement [22]. Unfortunately, quantitative evaluation cannot explain how and why a complex healthcare intervention was effective. To do this we interviewed a sub-sample of the trial participants, to see whether, how and why their initial beliefs about the causes, management and prognosis of chronic knee pain changed, after completing the programme.

Our premise was that participation on the programme would improve participants treatment belief in exercise as an effective intervention for chronic knee pain, and improve their confidence in their ability to exercise, thereby self-managing their problems.

## Methods

Detailed description of the RCT and rehabilitation programme are available [21]. Briefly, 418 participants aged 50 years of age or over, who had knee pain of more than 6 months duration, were identified from databases of 54 primary care surgeries in South East London and randomised to receive: i) usual care (control group, N = 140); ii) the ESCAPE-knee pain programme delivered to individual participants (N = 146); iii) the ESCAPE-knee pain programme delivered to small groups of 8 participants (N = 132). This report relates to the participants who undertook the rehabilitation programme, individually or in small groups.

### Rehabilitation programme

The rehabilitation programme consisted of 12 sessions (twice weekly for 6 weeks) supervised by a physiotherapist. To ensure consistency in content and delivery, the same physiotherapist supervised all the sessions for all participants. Each session comprised an education and exercise component;

#### Education component

The education aspect of the programme took place in a quiet room. The physiotherapist guided a 10-15 minute themed informal discussion designed to enhance patients' understanding of their condition its causes, consequences, prognosis and promote simple self-management and coping strategies by giving participants information, advice and teaching simple problem-solving and planning skills. It emphasised the importance of exercise/physical activity in reducing pain and improving function.

#### Exercise component

The exercises were performed in the gym of a physiotherapy out-patient department. The physiotherapist supervised each exercise session that lasted 30-45 minutes designed to increase strength, balance, co-ordination and confidence in collaboration with each participant taking into account their abilities. Initially 10 exercises were selected, but as the quantity and quality of exercise performance improved, these were progressed by omitting easier exercises and introducing more challenging exercises. In this way, each participant worked near their maximum capabilities in a controlled manner, with the physiotherapist nurturing participants' confidence in their ability to perform the exercises.

After 12 sessions participants were discharged with a home exercise regimen, advised to remain active and given information about local community exercise facilities, classes and self-help groups.

### Recruitment of interviewees

At the end of the baseline assessment of the RCT, participants were given a verbal outline of the qualitative study. As the qualitative study began after the RCT was underway, approximately a quarter of the patients in the RCT were approached, our target was to recruit 12 participants from each arm of the RCT using a purposive sample based on age, sex and ethnicity. People who expressed an interest in being interviewed received written information and their details were forwarded to the interviewer, who then contacted them explained the trial to them, addressed any concerns and, for those willing, arranged a baseline interview, when participant's signed consent was obtained.

The study complied with the Helsinki Declaration and had approval from the Local Research Ethics Committees of King's College, Guy's and St Thomas' (Ref No. 01-128) and Lewisham (Ref No. 01/10/17) Hospitals.

### Data collection and analysis

Interviews lasted 45-90 minutes and were conducted by the same interviewer in participants' homes or preferred location. It was emphasised to interviewees that the researchers running the RCT were unaware who was being interviewed and all transcripts would be anonymised and unattributable. Interview guides was used to ensure the project objectives were met and similar issues were explored with each participant (Appendix A), but interviewees were encouraged to raise, and the interviewers to explore, any issues considered relevant. Baseline interviews explored participants' experiences of living with knee pain, their understanding and beliefs about their condition, and strategies adopted to manage it. The same participants were re-interviewed after completing the programme to explore their experiences of the programme, its impact on their beliefs about knee pain and their views about the management of their condition (Appendix A). Preliminary analysis of the first few baseline and post-rehabilitation interviews identified emerging themes that influenced the content of subsequent interviews.

Interviews were audio taped, transcribed verbatim and imported into NVIVO v.2 software to organise and investigate the data. The thematic analysis was conducted. The interview transcripts were read several times by two researchers independently to familiarise themselves with the data, who met regularly to discuss and agree a coding scheme that assigned labels to segments of text identifying themes and resolve differences in coding and interpretation. Further codes were added as new themes emerged. All the authors received all interview transcripts, coded files and summaries of the emerging themes, reviewed and commented on the coding, analysis and plausibility of interpretation to refine and develop the analysis, and these issues were discussed at biannual project meetings. Cross-case and case-by-case analysis was undertaken to compare differences between participants' opinions and beliefs before and after rehabilitation.

## Results

Twenty nine participants were interviewed at baseline, twenty three agreed to be re-interviewed post-rehabilitation, and their characteristics and pseudonyms are shown in Table 1. The only difference between those who agreed to be re-interviewed and those who declined was that the three Black African interviewees declined to be re-interviewed.

**Table 1:** Details of interviewees with pseudonyms

Baseline Interviews				
	Trial arm	Age	Gender	Ethnicity
Number = 29	Individual = 12 Group = 17	Mean 67 yrs	Female = 19 Male = 10	Black Caribbean = 5 Black African = 3 Indian = 1 Caucasian = 20
**Pseudonym**				
Sandra	Individual	64	Female	Black Caribbean
Rachel	Individual	76	Female	Caucasian
Giles	Individual	76	Male	Caucasian
Donald	Group	67	Male	Caucasian
Barry	Group	71	Male	Indian
Veronica	Group	74	Female	Caucasian
Bernard*	*Individual*	*57*	*Male*	*Black African*
Geraldine	Group	65	Female	Caucasian
Doris	Group	71	Female	Caucasian
Ernest	Individual	60	Male	Black Caribbean
Derek	Individual	76	Male	Caucasian
Ruby*	*Individual*	*68*	*Female*	*Black Caribbean*
Robert	Individual	65	Male	Caucasian
Sue	Group	77	Female	Caucasian
Jacky	Group	61	Female	Caucasian
Cathy*	*Individual*	*62*	*Female*	*Caucasian*
Mary	Group	65	Female	Black Caribbean
Penny*	*Group*	*51*	*Female*	*Black African*
Jack	Group	72	Male	Caucasian
Betty	Group	84	Female	Caucasian
Lorraine	Group	65	Female	Black Caribbean
Harriet*	*Group*	*60*	*Female*	*Black African*
Martina	Group	53	Female	Caucasian
Alan	Group	58	Male	Caucasian
Monica	Group	59	Female	Caucasian
Celia	Group	60	Female	Caucasian
David*	*Individual*	*72*	*Male*	*Caucasian*
Stella	Individual	80	Female	Caucasian
Laura	Individual	66	Female	Caucasian
				
**Post-rehabilitation interviews**				
Number = 23	Individual = 8 Group = 15	Mean 68 yrs	Female = 15 Male = 8	Black Caribbean = 4 Black African = 0 Indian = 1 Caucasian = 18

*Participant declined to be re-interviewed

yrs - years

### Baseline interviews

**Figure 1:**
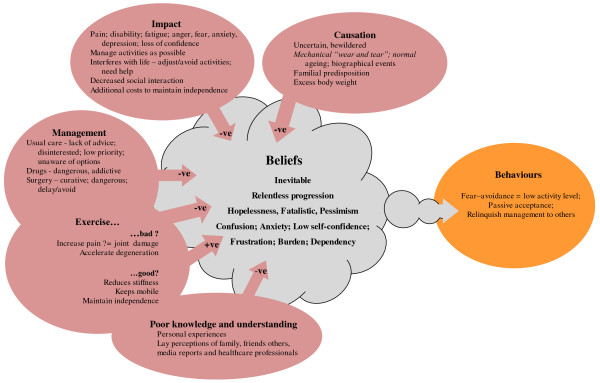
**Effects of causation and treatment beliefs and management experiences on people's initial beliefs and behaviours**.

Supporting quotations are given in Table 2 and a pictorial representation of how these affect health beliefs and subsequent behaviours in Figure 1.

**Table 2:** Themes and supporting quotations for interpretation of baseline interviews

Theme	Supporting Quote
***Causation:***	
- normal ageing	*"...I think it's just wear and tear. I think it's just accepted that you're going to get these things as you get older." Cathy*
- activities/injuries	*"...I was very athletic when I was young, and you know yourself with the athletes the injuries they get affects them later in life." Celia*
- hereditary	*"...My daughters have trouble with their knee now as well, don't know if it's inheritance...my granddad suffered with it, my mother's troubled with it." Sandra*
- excess body weight	*"...Well as I say, I need to lose weight...Well I mean there must be a lot of pressure on my knees as well, because I am overweight." Alan*
***Impact:***	
- pain - disability	*"...It's very tiring walking round shops...Some days I just sit here most of the time because its too painful too painful to move..." Rachel* *"...Getting out of bed, getting going, turning over in bed, waking up in the night...getting in and out of the car is a nightmare..." Geraldine* *"...I don't have a bath. I can't get up and down...I stand in the bath and wash down." Doris* *"...I'm good at going up stairs, it's the coming down I have difficulty with." Veronica* *"...I'm limited now, I can't go out as often as before, you know." Sandra* *"...It alters your life...it swings your life right round, it restricts you..." Deirdre*
- stoicism	*"...Well there's hardly a good day you know. I mean I just make the best of it. I don't try to you know, let it get me down. Although I have pain, I'll try and do what I can do you know rather than to just say 'I am in pain I cant do that'..." Ernest*
- need help	*"...my daughter has to be with me to have a bath...I can't move, I can't push my legs down from my knees, they won't function...I can't do shopping...my daughter does it all, yeah. You know, I mean she's ever so good to me...She does things, she does this of a morning before she goes to work..." Stella*
- psychosocial and emotional	*"... [inability to do gardening] makes me mad at times, cos I feel frustrated about it. I know there's masses of things that want to be done out there, I just haven't got the energy to do them...It makes you feel older." Rachel* *"...I'm very upset with myself cos, you know, when you're used to being mobile and able to do things for yourself, now you have to depend on people to do it, it's not very nice is it?...It's embarrassing. Like [at a dinner and dance] I sat down and had the dinner, and when I was to get up I couldn't move. I was so embarrassed and people looking at me." Mary*
***Management:***	
- limited advice	*"... [participants GP] never said anything, that's why I have always thought it's not worth bothering about. He's not bothered so I am not bothered..." Betty*
- medication	*"...I don't want too many tablets in me... I can try and bear pain myself." Sandra* *"...I do really try to keep off drugs because, you know, I mean I think that they all have side effects." Donald*
- surgery	*"I'm waiting for a knee replacement, cos I find it very difficult to get around, you know...I'm hoping the operation will correct it." Mary* *"...I'd have to be a lot worse than what I am now I think...well I mean if I've gone for thirty years I can go on for a few more." Veronica* *"...I don't want knee surgery, I've seen it happen; I've seen people have it very successfully and I've seen it be a disaster." Geraldine*
- non-pharmacological	*"...I get the pain and there is nothing that can be done about it..." Betty*
- exercise	*"...I'm questioning whether exercise might exacerbate or ease it. I really don't know." Donald* *"...I got a little bit frightened of doing exercise because I don't know what exercises will be detrimental to the knee or advantageous to it." Derek*
- weight control	*"...I think if you lose weight it keeps one off the tablets..." Geraldine*
***Prognosis: ***	
- hope	*"...I hope not permanent." Bernard*
- pessimistic	*"...I think probably it might get worse because it has been getting worse over the years." Mary*
- fatalistic	*"...I think, having to sit in one of those [wheelchairs]...I wouldn't want to do it, I don't want to get to that stage..." Rachel*
- surgical	*"...nothing will stop it getting worse I'm sure...if you have a replacement thing well alright that'll be alright but I mean otherwise they [his knees] just go on getting older..." Jack*

#### Causation

People were uncertain and bewildered about how, why and when their knee pain started. Most attributed knee pain to mechanical "wear and tear" of occupational, sporting and leisure physical activities, which led them to believe knee pain was an inevitable consequence of normal ageing. Some people tried to identify a specific incident as the start of their pain, but their recollection of the incident was often vague and they struggled to convince even themselves that this was the start of their problems. Frequently a familial predisposition for joint pain was mentioned with reference to a close relative, often female, who had "arthritis" or "rheumatism". Being overweight was also thought to cause or exacerbate pain.

#### Impact

##### Pain

Typically, people described episodic pain that increased gradually over several years. Pain varied greatly within and between participants, described variously as "a niggle", "not too bad", "murder" or "agony". Often weightbearing activities brought on pain, but people with advanced disease also had pain while sitting or in bed. The unpredictability of pain bewildered people.

##### Function limitations and disability

Pain, muscle weakness and fatigue after common activities of daily living impaired people's physical functioning and mobility. They stoically tried to carry out their normal activities, but often had to adjust or avoid activities (e.g. showering instead of bathing) in order to cope with their limitations and maximise their independence, and depended on family and friends for help with essential domestic and social activities (e.g. shopping, housework, gardening, transport, bathing).

##### Psychosocial and emotional

Limitations in functioning and the need for help made people feel frustrated, angry, depressed, embarrassed, incapacitated and a burden to others, and increased worries that the ultimate outcome would be severe disability, immobility and dependency.

#### Management

Our inclusion criteria meant all participants had consulted their GP about knee pain. People were often told the problem was due to wear and tear and getting old. Few could remember receiving information or advice, and they perceived knee pain was considered a benign condition that did not have a high priority, which confirmed their own beliefs and attitudes. No one had been offered a self-management programme. Management was seen as ineffectual, and consequently few were regularly consulting their GP despite on-going problems.

##### Medical

Management usually involved people being offered palliative medication. People used analgesia reluctantly, usually when pain was severe or before/after activities that exacerbated pain (e.g. shopping, gardening). They were concerned about side effects, becoming addicted and worried that taking it regularly would reduce its effectiveness. People taking medication for common co-morbidities (e.g. diabetes, cardiovascular, respiratory disease) wanted to limit the medications they were taking, preferring to omit analgesia and cope with pain rather than omit medication for co-morbidities seen as more serious, over which they had little control.

##### Surgical

People reasoned that while medication might alleviate symptoms, surgery was the only way to correct structural joint damage, eliminate pain and restore mobility, function and independence, but they wanted to delay surgery as long as possible. Others were more sceptical and frightened of surgery. Whether people held positive or negative expectations of surgery was strongly influenced by the exp **e**riences of family, friends, media reports or presence of co-morbidities that contra-indicated surgery.

##### Non-pharmacological

Awareness of treatment options other than medication and surgery was poor. Many people were using alternative remedies (e.g. fish oils, glucosamine, herbal remedies, acupuncture, osteopathy, copper bracelets, etc) on the recommendation of family, friends, media reports and advertisements. Some people found these helpful, others were sceptical but often continued to use them in the hope they would prevent or delay progression.

##### Exercise

Intuitively exercise and physical activity were perceived to keep people supple, mobile and maintain functioning. At the same time people associated activity with increasing pain, which they worried might accelerate joint damage. As a result of this confusion, and in the absence of any advice about what they should (not) be doing, few people were exercising and most were refraining from or avoiding activities.

##### Weight control

Often people who thought excess body weight increased stress on their joints, exacerbating pain and damage, tried dieting and exercising to lose weight, but activity-related pain impeded attempts to control their body weight.

#### Prognosis

People hoped rather than believed their symptoms would improve. They reasoned that joint damage was irreversible and likely to deteriorate without surgical correction. These beliefs arose from people linking the cause of joint pain to their biography, and the influence of other people's experiences and beliefs.

### Post-rehabilitation

**Figure 2:**
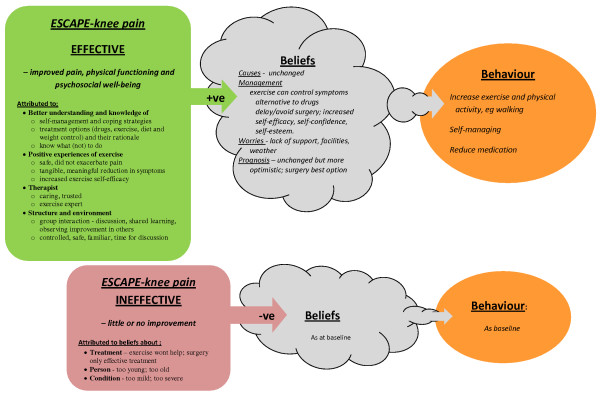
**Effect of ESCAPE-knee pain rehabilitation programme on people's health beliefs and behaviours**.

Supporting quotations are given in Table 3, with pictorial representation of how these affect health beliefs and subsequent behaviour in Figure 2.

**Table 3:** Themes and supporting quotations for interpretation of post-rehabilitation interviews

Theme	Supporting Quote
***Impact of programme:***	
- ineffective	*"...I was disappointed, because I hoped, I just hoped...but it didn't sort of do what I wanted it to do for my knees and I don't think anything will... I think your age, as you get older, you know, you get a bit dodgy." Doris*
- reduced pain	*"...the exercises we did at Dulwich were helping. and see I haven't had the pain... it was very helpful." Jack*
- improved function	*"...I felt generally strong, you know. Walking up stairs, I mean, at times I used to have to go up one step at a time, but then after the exercise I could just walk up the stairs and I was even beginning to try to walk normal..." Derek*
- improved psychosocial wellbeing	*"...Overall I have improved.... it's a feeling of general well being really... I feel a lot better in myself, I mean mentally, mostly mentally..." Rachel*
-return to normality	*"...If I can get myself back to a little bit of [line dancing] then I kind of umm, my life is kind of coming back to normality, you know, cause it can take over your life a bit as I say, you are scared of what to do and what not to do..." Celia*
***Reason for improvement:***	
- better knowledge and understanding	*"...I learned so much from [the physiotherapist]...I learnt about pain management..." Geraldine* *"...It helped me understand arthritis much better..." Jacky* *"...I class it as spring cleaning my mind..." Celia*
- exercise	*"... [helped understand] how to cope with pain...that exercise does help ease the pain and helps your mobility..." Jacky*
- allayed fears	*"...I didn't do no exercise, I didn't know I should do, I was frightened...but since I knew of the exercise, I have been doing it..." Josephine* *"...I thought if I exercise I am going to make the pain worse...they have showed me that I can still exercise even though I have a bad knee..." Alan*
- alternative self-management strategy	*"...I thought it was good, very good. To my mind I was helping to do something to help my knee pain..." Robert* *"...This [exercise] is much better because like I said I found is helpful, because I don't take any medicine..." Martina* *"...If you don't exercise you're never going to be able to manage the pain...Gentle exercise actually relieves the pain, and it means that you should be able to cut down [analgesia] and that the answer is not necessarily knee replacement..." Geraldine* *"...I mean exercise might stop it from getting worse any sooner that it would have done...before it deteriorates to the point where an operation might be needed..." Cathy*
- structure	*"...I had the opportunity to ask lots of questions..."Monica*
- group interaction and support	*"...you are all in there with similar problems, and it's the friendliness, like on a, personal level." Monica* *"...we formed very tightly knit group...we were all trying to help one another, you know." Celia*
- physiotherapist	*"...I think it's really a lot, in fact an enormous amount, to do with the facilitator, she's both kind of encouraging and yielding and nurturing and understanding, but also was able to use a bit of steel and get us off our bums, you know, so she's got those kind of qualities naturally..." Donald* *"... [Physiotherapist] gave us enormous confidence because she is such a, she is very very confident, obviously highly qualified, so it was good to have some body for an hour giving you good advice, which was sound..." Jack*
***Prognosis:***	
- optimistic	*"...I feel that I am not thinking about my knee pain anymore as a pain, I think about it more as preventing it by doing the exercises" Jack*
- incurable	*"... [arthritis]can ease...but there is no cure for it, so it's learning to live with it..." Jack*
- surgery	*"...I mean I feel better in myself, just a sense of well being but no won't be able to walk unless I have the surgery. It's just old age you see, and I get tired walking now so I think if I get them done I will be much better, I'll go on as much as I can doing the exercises..." Rachel*
***Future management:***	
- exercise at home	*"...I do the exercises upstairs when I get up in the morning... I find I might have a bit more time, because during the day you tend to let things slide." Doris*
- common activities	*"...I make sure I walk up and down the stairs during the day, cos that's one of the exercises you do..." Rachel* *"...I'm gonna exercise indoors. But if I walk I don't need much..." Sandra*
- join class	*"...Well I will just carry on with the umm, thingy bob [aqua aerobics], I can keep it under control. I am quite confident at the moment..." Alan*
- weight control	*"...If you are overweight it puts more pressure on your knee joints...if I lose weight, keep it under control, it won't get like real bad..." Alan*
- lack of support	*"...I think if there could be ongoing support in a group I'd feel positive..." Donald* *"...It would be nice to know if you were being naughty with your exercises you could ring them up and they just sort of say, right, get in here and you get in there for a couple of weeks or something to get you back into it." Celia*

#### Causation

In line with current understanding, the rehabilitation programme informed participants that the causes of chronic knee pain are often unclear, unidentifiable and multi-factorial, usually related to joint injury, previous activities, excess body weight and genetic predisposition. No attempt was made to identify the causes for individual participants' knee pain. Consequently, the programme did not alter participants' reasoning behind the cause of their pain.

#### Impact of the rehabilitation programme

##### Ineffective

Two participants were "disappointed" in the programme, experiencing little or no benefit from. This may have been a factor in the participants who withdrew from the study. The small number of people who found the programme ineffective makes it difficult to explore the reasons for ineffectiveness, but the two participants believed themselves too old or their symptoms too severe to enable them to benefit from exercise, and were sceptical and pessimistic about all interventions.

##### Reduced pain, improved function, ability

Most participants found the programme "interesting" and "informative", bringing "small" to "life-changing" improvements in pain, function (i.e. walking, domestic and social activities, getting on and off buses, driving). They felt less tired and had a general sense of better physical well-being.

##### Improved psychosocial well-being

The programme reduced anxiety and fear of activities people previously thought might increase pain, increased confidence in their ability to exercise safely and effectively, and generated a sense of self-achievement.

##### Return to normality

The physical and psychological improvements returned a degree of normality to people's lives. Some returned to previous activities they had begun to avoid for fear of harm, or take up activities to increase their level of physical activity.

#### Reasons for improvements

##### Increased knowledge and understanding

Receiving information and practical advice about what (not) to do, and the opportunity to discuss things that concerned and confused them with a healthcare professional, helped people appreciate their problems and what they could do to address these. In particular, they learned about the role of inactivity and excess body weight in development of knee pain, and how exercise and losing weight could control symptoms.

##### Exercise

Participation in the exercise regimen allayed people's fears, confusion and anxiety about the safety of exercise and showed them it was beneficial. Its successful completion convinced participants that exercise was an effective self-management strategy they were capable of implementing and a viable alternative to medication that might slow deterioration and delay or avoid surgery.

##### Structure

People appreciated being able to discuss and assimilate information without the time constraints of medical consultations. Participants, especially elderly participants, felt familiar comfortable and at ease in the hospital environment. Although the clinical benefits people derived from the rehabilitation programme were similar regardless of whether it was received individually or in small groups [21], participants who received group-rehabilitation thought meeting, sharing experiences and the support derived from fellow group members was beneficial, and that observing improvements in others was a source of encouragement.

##### Patient-physiotherapist partnership

The care, support and guidance participants received during the informal discussions helped build a trusting, collaborative partnership between patient and physiotherapist. This increased participants' confidence and trust in the physiotherapist and belief in the rehabilitation programme. The interpersonal qualities and professional skills of the supervising therapist were considered as important to the success of the programme as the content of the programme itself.

#### Future management

People accepted exercise wouldn't eliminate their problems, but they understood how they could use it to manage their problems better. They were planning to continue their home exercise programme and use common physical activities (walking, gardening, housework, leisure activities) as informal exercise. Many were concerned their efforts might be thwarted by worries about personal safety, lack of facilities or environmental issues such as inclement weather. However, their greatest concern was losing the ongoing support of the physiotherapist would undermine their motivation to exercise, and they expressed a desire for on-going support.

#### Prognosis

People continued to mention the incurability of knee pain, thought deterioration was likely and that surgery was still considered the most effective way to improve pain and function. However, increased understanding, positive experiences of exercise and influence of the physiotherapist engendered greater optimism about the prognosis, and generally people felt better able to cope with their problems.

## Discussion

These interviews document the initial health beliefs and expectations of people with chronic knee pain, and how and why these changed following participation on *ESCAPE-knee pain *- a rehabilitation programme that integrated patient education, self-management and coping strategies and exercise. For most participants, pessimistic, negative beliefs and expectations were turned into a more optimistic positive outlook, with a greater appreciation of treatment options through the structure, content and delivery of the programme, in particular by improving their knowledge, understanding and beliefs in the safety, importance and value of exercise in the management of knee pain. They confirmed the programme's theoretical premise that improvements are effected by altering people's treatment beliefs about exercise and exercise self-efficacy.

Our participants shared the generally held unhelpful beliefs that chronic knee pain is a normal, inevitable, untreatable consequence of life and ageing, best treated by surgery and might be exacerbated by activity [5-10,23]. Unfortunately, such beliefs lead to passive acceptance of an inexorable increase in pain and disability, deter people from seeking treatment, undermine confidence and adherence to medication and active self-management strategies, such as exercise, and encourage people to relinquish responsibility for its management to others [24].

Attaining behavioural change, for example regular exercise, requires people to believe that the behaviour is safe, necessary and beneficial [25], that they have the ability to accomplish it [26-28] and that the benefits will outweigh any time, effort and resource costs [25]. Concerns about exercise causing joint damage are not challenged during usual primary care management, nor are the necessity and potential benefits of exercise explained and emphasised. Consequently, most people were not exercising, even though many considered exercise would be good for their health, function and mobility. The education component of *ESCAPE-knee pain *confirmed people's positive beliefs about exercise, convincing them of the importance, safety and benefits attainable through exercise.

However, simply giving people information about their condition and the benefits of "good" behaviours is not usually sufficient stimulus to effect behavioural change - especially if the behaviour is burdensome, people are uncertain they can perform it, are unconvinced about its effectiveness and have concerns that it may harm them [29,30]. Experiencing a successful intervention restructures people's treatment beliefs about the management of their problems [20]. The results of the RCT showed that the *ESCAPE-knee pain *programme improved people's exercise beliefs and self-efficacy [21,31]. This qualitative study suggests these improvements reflect people's understanding and belief in exercise as an effective self-management strategy that they have the ability to implement.

Participants considered supervision essential for enhancing helpful exercise beliefs and self-efficacy. This raised concerns that without continued support their motivation to exercise would wane [27,32-38]. Thus the programme may be creating reliance on supervision as a requirement for exercise, which would undermine people's exercise self-efficacy and the usefulness exercise as a self-management strategy [23,36,37,39,40] if continued supervision were required to maintain the benefits. However, *ESCAPE-knee pain *is equally effective whether delivered to groups of participants or individual participants [21]. Therefore group rehabilitation could be adapted so that continued contact with group members might provide the encouragement, support and motivation required to promote continued participation in regular exercise.

The strengths of this study are its size and longitudinal design. It is a large qualitative study nested in a large pragmatic clinical trial. It is the first study to document the same people's beliefs before and after a rehabilitation programme of this nature. The similarity of our participant's baseline perceptions of their condition, treatment and prognosis with those reported in previous studies [5-10], suggest our participants are a representative patient population. These factors allow us to confidently infer that the improvements reported were due to the rehabilitation programme, and will generalise to the wider patient population.

Potential limitations are subjectivity and bias. First, we could not objectively verify participants' self-reported exercise behaviour. They might have overstated their improvement to justify their investment of time and effort, to please the interviewer and/or to reflect well on themselves and the therapist. We tried to minimise the effect of these potential problems by interviewing people in their homes and emphasising anonymity to foster frankness and honesty. Nevertheless, because the interviews focused on participants' experiences on the rehabilitation programme, participants may have been unable to distinguish between the studies. Second, the interview guide was devised by the researchers and could have influenced the findings, although the interviewer did probe other issues that arose and encouraged people to discuss whatever they felt pertinent. Third, interpretation is context-specific and these findings are only one interpretation of people's perspectives of their disease, its management and the views of the rehabilitation programme. Finally, "volunteer bias" may have led to the recruitment and retention of people very enthusiastic about exercise, generating a deceptively positive perspective of the programme, although the baseline interviews suggest our participants held similar views as participants of other studies and were not over-enthusiastic.

## Conclusion

Current management of chronic knee pain in primary care tacitly confirms erroneous, negative beliefs about the cause, treatment and prognosis of chronic knee pain, and fails to foster intuitive positive beliefs about the benefit of exercise - it accentuates the negative and eliminates the positive. Our prospective interviews of participants undertaking an integrated rehabilitation programme for chronic knee pain explain why the programme was successful in reducing pain, improving function and psychosocial well-being. Advice about the importance of exercise and active participation on an exercise-based rehabilitation programme reduces the symptoms and consequences of chronic knee pain and gives people an alternative self-management strategy. Altering treatment beliefs are vital in altering behaviour [19,23,25,27,36] and more important than altering beliefs about causation and prognosis, and improvement in physiological variables [22].

## Appendix A

### Baseline Interview Guide

When did you start having knee pain?

Why do you think it started?

What makes it better/worse?

What do you do to avoid or reduce pain?

Why do you think this works?

Who have you seen about your knee pain?

*(If seen) *Did they give you some advice or information? Did you find this helpful? Are you following that advice?

How do you manage to get things done?

Does your knee pain stop you doing anything?

*(If limited) *How do you feel about not being able to do those things?

What treatment have you received?

*(If received treatment) *How effective was it? Are you still doing it? Why (not)?

Are you aware of any other treatments for knee pain?

*(If aware) *Have you considered them? Why (not)?

What are your feelings about exercise?

What do you think will happen with your knee pain in the future?

Do you think your knee pain will ever go away?

### Post-rehabilitation interview guide

How has your knee pain been?

*(If changed) *When did you notice this change? What do you think caused the change?

Last time you thought your knee pain was caused by...*(previous reason)..*.do you still think that?

You mentioned you had difficulty...*(previous problems)..*.How are things now?

*(If changed) *How do you feel about the (lack of) change? Is there anything you do now to help you control your knee pain? Do you still get help from others?

Are you still...*(e.g. taking medication, using other strategies)..*.to help reduce pain?

Since we spoke you have been on the rehabilitation programme. Can you tell me about that?

Did it help? How? Why (not)? What was good/bad about it?

Do you think you will keep doing the things...*(regular physical activity, weight control)..*.suggested?

How easy or difficult do you think it will be to continue? What might prevent you? What might help you?

Have you felt changes to other areas of your life...*(e.g. social life/relationships)*?

What do you think will happen with your knee pain in the future?

## Competing interests

The authors declare that they have no competing interests.

## Authors' contributions

MH conceived the study, participated in its design and coordination, read all interview transcripts, commented on the coding, analysis and interpretation, prepared drafts and final manuscript. NW contributed to the conception of the study, commented on its design, helped identify potential participants, commented on the drafts of the manuscript. VB recruited and consented all participants, carried out all the interviews, read all transcripts, performed the coding schemes, contributed to interpretation of the data, prepared the initial draft manuscript. NB contributed to the design of the study, read some interview transcripts, commented on the coding, analysis and interpretation, commented on the drafts of the manuscript. FS participated in the design of the study, read all interview transcripts, performed the coding schemes and analysis, interpreted the data, commented on the drafts of the manuscript. All authors read and approved the final manuscript.

## Pre-publication history

The pre-publication history for this paper can be accessed here:

http://www.biomedcentral.com/1471-2474/11/31/prepub
